# Isolation of a Monoclonal Human scFv Against Cytomegalovirus pp71 Antigen Using Yeast Display

**DOI:** 10.3390/antib14030057

**Published:** 2025-07-10

**Authors:** Kazuhisa Aoki, Rikio Yabe, Sayaka Ono, Mayumi Saeki, Yuri Tanno, Hidetaka Tanno

**Affiliations:** 1 Cancer Immunology Project, Tokyo Metropolitan Institute of Medical Science, 2-1-6 Kamikitazawa, Setagaya-ku, Tokyo 156-8506, Japan; 2 Graduate School of Medical and Dental Sciences, Institute of Science Tokyo, Bunkyo-ku, Tokyo 113-8510, Japan

**Keywords:** yeast surface display, cytomegalovirus, pp71, scFv

## Abstract

Background: Human cytomegalovirus (CMV) is a major pathogen that poses significant risks to immunocompromised individuals and neonates. The tegument protein pp71, encoded by the UL82 gene, plays a pivotal role in initiating viral lytic replication and evading host immune responses. Despite its clinical relevance, standardized monoclonal antibodies (mAbs) for pp71 remain limited, prompting the need to expand the available repertoire of antibodies targeting this critical protein. Methods: In this study, we constructed a diverse human single-chain variable fragment (scFv) library using RNA derived from the B cells of four healthy donors. The library was expressed in *Saccharomyces cerevisiae*, and iterative rounds of magnetic-activated cell sorting (MACS) were performed against recombinant pp71. Clonal enrichment was monitored using flow cytometry. Results: Among the isolated clones, one designated ID2 exhibited high sensitivity and specificity for pp71, as demonstrated by flow cytometry, immunofluorescence, an enzyme-linked immunosorbent assay (ELISA), and biolayer interferometry (BLI). Conclusions: Collectively, these findings establish a novel pp71-specific mAb and underscore the utility of yeast surface display combined with MACS for expanding the antibody toolkit available for CMV research and diagnostics.

## 1. Introduction

Human cytomegalovirus, a member of the *Herpesviridae* family, infects a great majority of people worldwide; a recent meta-analysis estimated global IgG seroprevalence at ~83%, with prevalence varying markedly by region and socioeconomic status [1,2]. Although most immunocompetent individuals experience asymptomatic or mild infections, CMV can cause severe disease in fetuses, neonates, and immunocompromised individuals such as transplant recipients and patients with AIDS [3,4,5]. In these high-risk groups, CMV activation often leads to life-threatening conditions, including pneumonia, retinitis, and systemic disease, underscoring the importance of enhanced diagnostic and therapeutic strategies.

The CMV genome encodes numerous proteins, among which pp71, encoded by the UL82 gene, has been recognized as a pivotal tegument protein that stimulates immediate-early (IE) gene expression and facilitates the initiation of lytic replication [6,7]. Furthermore, pp71 can disrupt host immune surveillance via the downregulation of MHC class I molecules [8]. These functions make pp71 an attractive target for studying CMV pathogenesis and for developing potential diagnostic tools or antiviral strategies. However, despite its critical role in CMV infection, progress in diagnostics and basic research is hampered by the scarcity of commercially available monoclonal antibodies (mAbs) against pp71. Although leading academic groups in CMV research have provided pp71-specific mAbs as kind gifts for research purposes [9,10,11], there remains a need to expand the repertoire of antibodies targeting this essential protein.

Monoclonal antibodies (mAbs) are integral to biomedical research and clinical diagnostics due to their high specificity for a single epitope [12]. However, conventional hybridoma-based approaches for mAb generation are often time-consuming, labor-intensive, and dependent on animal immunization. In contrast, display technologies such as phage display, ribosome display, and yeast surface display enable the high-throughput screening and optimization of antibody fragments [13]. Among these, yeast surface display offers notable advantages. *Saccharomyces cerevisiae* provides a eukaryotic secretory apparatus that can promote oxidative protein folding and certain post-translational modifications (e.g., N-linked glycosylation), which may help in generating properly folded and functionally active antibody fragments [14,15]. Additionally, the isolation of high-affinity clones via fluorescence-activated cell sorting (FACS) is both quantitative and efficient.

Here, we report the isolation of a pp71-specific monoclonal antibody using a yeast-displayed single-chain variable fragment (scFv) library. We performed iterative selection rounds against recombinant pp71, followed by the characterization of the resulting clones using immunoassays (flow cytometry, immunofluorescence, ELISA, and BLI analysis). Our findings confirm the high specificity and affinity of the isolated mAb for pp71, thereby providing a novel tool for investigating CMV biology and facilitating the development of diagnostic and therapeutic approaches targeting this essential viral protein.

## 2. Materials and Methods

### 2.1. Construction of Random Human scFv Display Yeast

Peripheral blood mononuclear cells (PBMCs) from four healthy human donors were purchased from Lonza (#CC-2704). Total B cells were isolated from the PBMCs using part of an IgG+ Memory B Cell isolation kit (Miltenyi Biotec, Bergisch Gladbach, Germany, #130-094-350) and an AutoMACS Pro Separator (Miltenyi Biotec). The isolated total B cells were seeded at a density of 1.83 × 10^4^–6.54 × 10^4^ cells/mL in a 48-well plate and cultured for five days in 0.5 mL per well of ImmunoCult Human B Cell Expansion Kit medium (STEMCELL Technologies, Vancouver, BC, Canada, #ST-100-0645). The number of B cells used in this experiment is described in Supplementary Table S1.

The expanded B cells were lysed in TRIzol™ Reagent (ThermoFisher Scientific, Waltham, MA, USA, #15596026), and RNA was extracted. Reverse transcription was performed using SuperScript™ IV Reverse Transcriptase (ThermoFisher Scientific, #18090010). The VH and VL regions were then amplified separately via PCR with cDNA, custom multiplex primers, and Platinum SuperFi II Green PCR Master Mix (ThermoFisher Scientific, #12369010). After gel extraction using NucleoSpin^®^ Gel and PCR Clean-up (Takara, Shiga, Japan, #U0609C), random mutations were introduced into the amplicons using a Diversify PCR Random Mutagenesis Kit (Takara, #630703).

After gel extraction, to facilitate assembly, a complementary linker sequence for joining VH and VL was appended to each amplicon via a second PCR using custom primer sets and Q5 Hot Start High-Fidelity 2X Master Mix (NEB, Ipswich, MA, USA, #M0494L). These linker-tagged VH and VL fragments were gel-purified, mixed at equimolar ratios, and assembled into a full-length scFv using NEBuilder HiFi DNA Assembly Master Mix (NEB, #E2621L). After column purification was performed (Takara, #U0609C), adaptor sequences that overlapped with the pCTCON2 plasmid were added to the scFv amplicon via PCR, and then gel was extracted. The expression plasmid pCTCON2 was purchased from Addgene (Plasmid, Watertown, NY, USA, #41843) and cut using the restriction enzymes SalI (NEB, #R3138S), BamHI (NEB, # R3136S), and NheI (NEB, R3131S), and then gel was extracted. Finally, 6 μg of the cut pCTCON2 and 18 μg of the scFv amplicon were mixed and then electroporated into 1.6 × 10^9^ AWY101 yeast cells, as previously reported [16] (AWY101 was a kind gift from Eric V. Shusta, University of Wisconsin–Madison). Through homologous recombination in *S. cerevisiae*, the scFv insert was integrated immediately downstream of the bidirectional GAL1/GAL10 promoter in the pCTCON2 vector, enabling the galactose-inducible expression of the Aga2–scFv fusion protein. The electroporated yeasts were cultured in SDCAA medium (20 g/L dextrose, 6.7 g/L Difco yeast nitrogen base w/o amino acids (BD, Franklin Lakes, NJ, USA, #291940), 5 g/L acid hydrolysate of casein (BIOKAR Diagnostics, Paris, France, #A1404HA), 5.4 g/L Na_2_HPO_4_, and 9.68 g/L NaH_2_PO_4_·2H_2_O) for two days. After washing the yeasts once with SGDCAA medium (2 g/L dextrose, 20 g/L galactose, 6.7 g/L Difco yeast nitrogen base w/o amino acids, 5 g/L acid hydrolysate of casein, 5.4 g/L Na_2_HPO_4_, and 9.68 g/L NaH_2_PO_4_·2H_2_O), they were cultured in SGDCAA at 20 °C for two days for the induction of surface antibodies. All SDCAA and SGDCAA media in this study contained penicillin–streptomycin (Wako, Osaka, Japan, #168-23191). The custom multiplex primer sequences used in this study are available from the corresponding author upon reasonable request.

### 2.2. Pp71 Expression and Purification

A DNA encoding Flag-pp71-His-SBP (where SBP denotes the streptavidin-binding peptide tag) was synthesized by Twist Bioscience (South San Francisco, CA, USA) and cloned into pcDNA3.1 (ThermoFisher Scientific, #V79020). The plasmid was transfected into Expi293F cells according to the manufacturer’s protocol (ThermoFisher Scientific, #A14635). After 7 days of culture, the cells were harvested and washed twice with PBS. The cell pellet was lysed in ice-cold lysis buffer (50 mM phosphate buffer, pH 7.5, 300 mM NaCl, 20 mM imidazole, 0.1% Igepal CO-630, 5 mM MgSO_4_, and 1x Pierce Protease Inhibitor Tablet, EDTA-free (ThermoFisher Scientific, #A32965)). The lysate was incubated on ice for 30 min with occasional mixing, followed by centrifugation at 17,000× *g* for 15 min at 4 °C. The supernatant was incubated overnight at 4 °C with Ni-NTA agarose resin (ThermoFisher Scientific, #88221) under gentle rotation. The resin was washed with washing buffer (50 mM phosphate buffer, pH 7.5, 300 mM NaCl, 50 mM imidazole) three times. The bound protein was eluted with elution buffer (50 mM phosphate buffer, pH 7.5, 300 mM NaCl, 250 mM imidazole). The eluate was concentrated and buffer-exchanged to PBS using Amicon Ultra-15 centrifugal filter units with a 10 kDa molecular weight cutoff (Merck, Darmstadt, Germany, #UFC901024). The purity of Flag-pp71-His-SBP was validated using SDS-PAGE, followed by CBB staining (Supplementary Figure S1). The concentration of the purified Flag-pp71-His-SBP protein was determined using a DeNovix DS-11+ spectrophotometer (Wilmington, NC, USA).

### 2.3. MACS

The optical density (OD_600_) of the yeast culture was measured, and, assuming that an OD_600_ of 1 corresponds to ~1 × 10^7^ cells/mL, cells equivalent to 2 × 10^9^ yeast cells were washed once with rinsing buffer (Miltenyi Biotec, AutoMACS Rinsing Solution #130-091-222 supplemented with MACS^®^ BSA Stock Solution #130-091-376). The yeasts were incubated with 4.6 μg of Flag-pp71-His-SBP at room temperature for one hour. After washing the yeasts twice with rinsing buffer, they were mixed with Anti-His-tag mAb-Alexa Fluor 647 (MBL, Tokyo, Japan, #D291-A64) and incubated for 30 min at 4 °C with rotation. Then, the yeasts were washed twice using the rinsing buffer and mixed with Anti-Cy5/Anti-Alexa Fluor 647 MicroBeads (Miltenyi Biotec, #130-091-395). After 15 min of rotation at 4 °C, the yeasts were washed once with rinsing buffer, and the magnetic separation Posseld program was performed using AutoMACS pro. The positive fraction was washed once with SDCAA medium and then cultured in SDCAA medium for one or two days at 30 °C, depending on the concentration of the yeasts. Following yeast expansion, the cells were washed once with SGDCAA medium and cultured in SGDCAA medium for two or three days at 20 °C. This screening process was repeated, with reagents alternated in each subsequent round. Specifically, the Anti-His-tag mAb-Alexa Fluor 647 and Anti-Cy5/Anti-Alexa Fluor 647 MicroBeads were replaced with FITC anti-His-Tag Antibody (BioLegend, San Diego, CA, USA, #362618) and Anti-FITC MicroBeads (Miltenyi Biotec, #130-048-701), respectively. MACS separation was performed up to round 5. From round 2 onward, 2.3 μg of Flag-pp71-His-SBP was used instead of 4.6 μg to enrich for higher-affinity clones.

### 2.4. Flow Cytometry Analysis of scFv-Expressing Yeasts

To evaluate pp71 binding to scFv-expressing yeasts, approximately 1 × 10^6^ yeast cells (based on OD_600_ measurement) were washed once with rinsing buffer and then incubated with 0.1 μg of Flag-pp71-His-SBP for 30 min at 4 °C. After washing twice with rinsing buffer, the yeasts were incubated with FITC-streptavidin (BioLegend, #405202, 1:200) to detect the SBP tag on pp71 bound to the yeast surface and with Anti-Myc-tag mAb-Alexa Fluor 647 (MBL, #M047-A64, 1:100) to visualize the C-terminal Myc epitope of the surface-displayed scFv. After washing three times with rinsing buffer, the yeasts were analyzed using a FACSCantoII analyzer (BD Biosciences, San Jose, CA, USA) and FlowJo v10.

### 2.5. scFv-Fc Expression and Purification

Round 5 yeasts were spread on an SDCAA plate, and single colonies were picked up and cultured in SDCAA medium followed by the induction of the scFv in SGDCAA medium to assess Flag-pp71-His-SBP binding via flow cytometry. After the flow cytometry analysis, we isolated plasmid DNA using a Zymoprep Yeast Plasmid Miniprep Kit II.

In parallel, DNA coding a secretion signal peptide and the BALB/c mouse IgG1 Fc region sequence was synthesized by Twist Bioscience and cloned into pHEK293 Ultra Expression Vector I (Takara, #3390). This vector backbone was then PCR-linearized using PrimeSTAR Max DNA Polymerase Ver. 2 (Takara, #R047B) with primers designed to generate ends matching the signal peptide sequence at one terminus and the GS-linker–Fc junction at the other. The scFv region was PCR-amplified from the yeast plasmids with HA- and Myc-specific primers. The HA primer overlapped the signal peptide region, and the Myc primer overlapped the GS-linker–Fc region. Finally, the HA-scFv-Myc insert and PCR-linearized backbone were assembled using NEBuilder HiFi DNA Assembly Master Mix, yielding the final HA-scFv-Myc–Fc expression construct. Following assembly, the HA-scFv-Myc–Fc construct was transformed into *E. coli* (STBL3), and plasmid DNA was isolated using miniprep. Clone identity was confirmed via Sanger sequencing using vector-specific primers annealing to the pCTCON2 backbone and an internal primer annealing to the Fc region.

The plasmids were transfected into Expi293F cells according to the manufacturer’s protocol. One week after the transfection, the supernatant was filtered using Millex-HP 0.45 μm (Merck, #SLHPR33RS) and then incubated with Ab-Capcher beads (ProteNova, Kagawa, Japan, #P-002-10) at 4 °C overnight. The beads were washed with PBS three times, then eluted with Glycine-HCl pH 2.8 three times, and immediately neutralized with 1M Tris-HCl pH 8.0. Buffer was exchanged to PBS using Amicon Ultra-15 centrifugal filter units with a 10 kDa molecular weight cutoff (Merck, #UFC901024). The scFv-Fcs concentrations were quantified using a DeNovix DS-11+ spectrophotometer.

The scFv-Fc sequences used in this study are available from the corresponding author upon reasonable request.

### 2.6. Flow Cytometry Analysis Using scFv-Fcs

pp71-Flag-His or pp71-V5 plasmids were transfected into HEK293FT cells using PEI max. Two days after transfection, the cells were fixed and permeabilized with a FOXP3 Fix/Perm Buffer Set (BioLegend, #421403). The cells were incubated with the ID1 or ID2 scFv-Fc (1.3 μg/mL) and then stained with FITC anti-His tag antibody (BioLegend, #362618, 1:100) or Alexa Fluor 488 V5 Tag Monoclonal Antibody (Invitrogen, #37-7500-A488, 1:200) and APC anti-HA.11 tag antibody (BioLegend, #901524, 1:100). The cells were analyzed with a FACSCantoII analyzer (BD Biosciences).

### 2.7. Sandwich ELISA

Anti-DYKDDDDK tag antibody (1 μg/mL; Clone L5; BioLegend, #637319) was precoated on MaxiSorp 96-well plates (ThermoFisher Scientific, #442404) at 4 °C overnight. The plates were washed with PBS/0.05% Tween20 and blocked with 10% FBS/PBS at room temperature for 1 h. After washing with PBS/0.05% Tween20, 0.125, 0.25, 0.5, and 1 μg/mL of Flag-pp71-His-SBP or BSA (Globulin-free; NACALAI TESQUE, Kyoto, Japan, #01281-26) in triplicate, the wells were incubated at room temperature for 2 h. After washing with PBS/0.05% Tween20, the wells were incubated with 5 μg/mL scFv-Fc (ID 1 or ID2) at room temperature for 1 h, followed by HRP-conjugated goat anti-mouse IgG (Clone Poly4053; 1:5000 dilution; BioLegend, #405306) at room temperature for 1 h. ELISA POD Substrate TMB solution (Easy) (NACALAI TESQUE, #05299-54) was added and stopped with 1 N HCl (NACALAI TESQUE, #18322-95). The absorbance at a dual wavelength of 450/595 nm was measured using a microplate reader, ARVO X3 (PerkinElmer, Shelton, CT, USA).

### 2.8. BLI Analysis

BLI binding assays were performed in 96-well black microplates (Sigma Aldrich, St. Louis, MO, USA, #781608) at 30 °C with shaking at 1000 rpm using an Octet R4 instrument (Sartorius, Göttingen, Germany) and anti-penta-His (HIS1K) biosensors (Sartorius, #18-5120). Flag-pp71-His-SBP was diluted to 5 µg/mL in assay buffer (PBS supplemented with 0.005% Tween20 and 0.02% BSA). ID1 and ID2 scFv-Fc were diluted to 66.7 nM (6.4 µg/mL) in assay buffer. For the ID2 antibody, 2-fold serial dilutions (1.04–16.7 nM) were prepared to determine the equilibrium dissociation constant.

Binding experiments consisted of the following steps: First Baseline, assay buffer for 60 s; Loading, 5 µg/mL His-tagged pp71 for 900 s; Second Baseline, assay buffer for 60 s; Association, analyte (antibodies) for 600 s; and Dissociation, assay buffer for 300 s. The resulting association curve (0–600 s) and disassociation curve (0–300 s) were analyzed using the Octet Analysis Studio 12.2.2.26 software (Sartorius). The data were fitted with a 1:1 binding model to obtain K_D_.

### 2.9. Immunofluorescence

Flag-pp71-His-SBP or V5-pp71 plasmids were transfected into HEK293FT cells using PEI max. The following day, the cells were seeded onto coverglasses in 6-well culture plates. Three days post-transfection, the cells attached to the coverglasses were fixed with 4% paraformaldehyde for 20 min at room temperature, followed by treatment with 1% Igepal CO-630 in PBS for 10 min at room temperature. The cells were then blocked with 3% BSA in PBS for 1 h at 37 °C. Subsequently, the cells were incubated with ID2 scFv-Fc (0.5 μg in 150 μL of 3% BSA in PBS) for 1 h at 37 °C. For the cells transfected with the Flag-pp71-His-SBP plasmid, staining was performed with Anti-His-tag mAb-Alexa Fluor 594 (MBL, #D291-A59, 1:250) and Alexa Fluor 488 anti-HA.11 Epitope Tag Antibody (BioLegend, #901509, 1:500). For the cells transfected with the V5-pp71 plasmid, staining was carried out with Alexa Fluor 594–anti-HA tag antibody (MBL, #M180-A59, 1:100) and Alexa Fluor 488 V5 Tag Monoclonal Antibody (ThermoFisher Scientific, #37-7500-A488, 1:1500) for 1 h at 37 °C. The coverglasses were mounted with VECTASHIELD Antifade Mounting Medium with DAPI (VECTOR LABORATORIES, Newark, NJ, USA, #H-1200) and observed using a ZEISS LSM980 confocal microscope. Images were acquired and processed using the ZEN 3 software (Zeiss, Oberkochen, Germany).

## 3. Results

### 3.1. Construction of Human scFv Library and Yeast Display Screening

**Figure 1:**
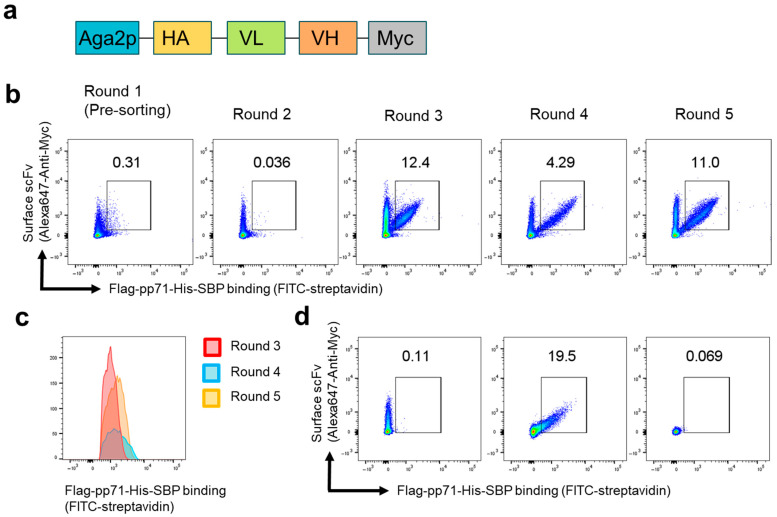
Screening of anti-pp71 human scFv using yeast display technology. (**a**) The diagram illustrates the structure of the scFv expression pCTCON2 plasmid used for yeast display. The scFv is expressed as a fusion protein with agglutinin subunit 2 (Aga2p), which anchors the protein to the yeast cell surface. HA, hemagglutinin epitope tag; Myc, c-Myc epitope tag; VL, variable light chain; VH, variable heavy chain. (**b**) Flow cytometry analysis of Flag-pp71-His-SBP binding to yeast cells in each round of selection. (**c**) Representative histograms showing the signal intensity of Flag-pp71-His-SBP binding to yeast cells from rounds 3 to 5. (**d**) Representative flow cytometry plots showing Flag-pp71-His-SBP binding to yeast cells derived from single colonies in round 5.

First, we constructed a human scFv library using RNA derived from the B cells of four healthy donors. The scFv library was cloned into the pCTCON2 yeast expression plasmid [17], designed to express an Aga2-HA-VL-linker-VH-Myc fusion protein (Figure 1a). The constructed scFv library was electroporated into the *Saccharomyces cerevisiae* strain AWY101 [18]. After expansion in SDCAA medium, scFv expression was induced by culturing the yeast in SGDCAA medium.

In parallel, we transfected Expi293F cells with a plasmid encoding Flag-pp71-His-SBP. The recombinant Flag-pp71-His-SBP protein was purified and used for yeast display screening.

The Flag-pp71-His-SBP protein was combined with yeast cells, and then MACS was performed. After expanding and inducing the isolated yeast cells, the sorting process was repeated three additional times.

To evaluate scFv expression and the antigen binding of each round of yeasts, we performed a flow cytometry analysis on the induced yeast cells. The yeast cells were incubated with Flag-pp71-His-SBP, and then cell surface scFv and bound Flag-pp71-His-SBP were stained. As shown in Figure 1b, the proportion of antigen-bound yeast cells increased from less than 0.31% in round 1 to 12.4% in round 3.

However, binding proportions fluctuated across selection rounds. In round 1—the naive, unselected library—non-specific binding was relatively high, likely reflecting the large diversity of low-affinity variants that was reduced after the first enrichment. In round 4, the antigen-positive fraction fell to 4.29% under identical selection conditions; nevertheless, the median FITC-streptavidin fluorescence intensity of this population exceeded that of round 3. This increase is consistent with either enrichment of higher-affinity scFv clones or enhanced scFv surface display. A similar elevation in fluorescence intensity was observed in round 5 (Figure 1c).

To isolate individual scFv plasmids, round 5 yeast cells were plated on SDCAA agar to obtain single colonies. After colony formation, 12 single colonies were picked and cultured in SDCAA medium and then transferred to SGDCAA medium to induce scFv expression. The yeast cells were subsequently incubated with Flag-pp71-His-SBP, stained for cell surface scFv and bound Flag-pp71-His-SBP, and analyzed using flow cytometry. Among the 12 single-colony isolates, 3 clones expressed surface scFv but failed to bind Flag-pp71-His-SBP; a representative flow cytometry plot for 1 of these clones is shown in Figure 1d (left). Plasmid DNA from one of these clones was recovered, Sanger-sequenced, and designated ID1, which was used as a negative control scFv. Four clones expressed surface scFv and bound Flag-pp71-His-SBP; Figure 1d (center) shows a representative plot for one of them. Sanger sequencing confirmed that all four clones shared an identical scFv sequence, hereafter referred to as ID2, which was selected for further characterization. The remaining five clones neither expressed scFv nor bound the antigen; a representative plot is shown in Figure 1d (right). The flow cytometry analysis of the 12 clones is detailed in Supplementary Figure S2.

### 3.2. Characterization of ID2

Next, we PCR-amplified clones ID1 and ID2 using primers specific to the HA and Myc regions in order to generate full-length HA-scFv-Myc fragments (Figure 1a). The resulting amplicons were cloned into a customized plasmid harboring a mouse IgG1 Fc region for scFv-Fc fusion protein expression. These scFv-Fc plasmids were then transfected into Expi293F cells, and the expressed scFv-Fcs were successfully purified.

**Figure 2:**
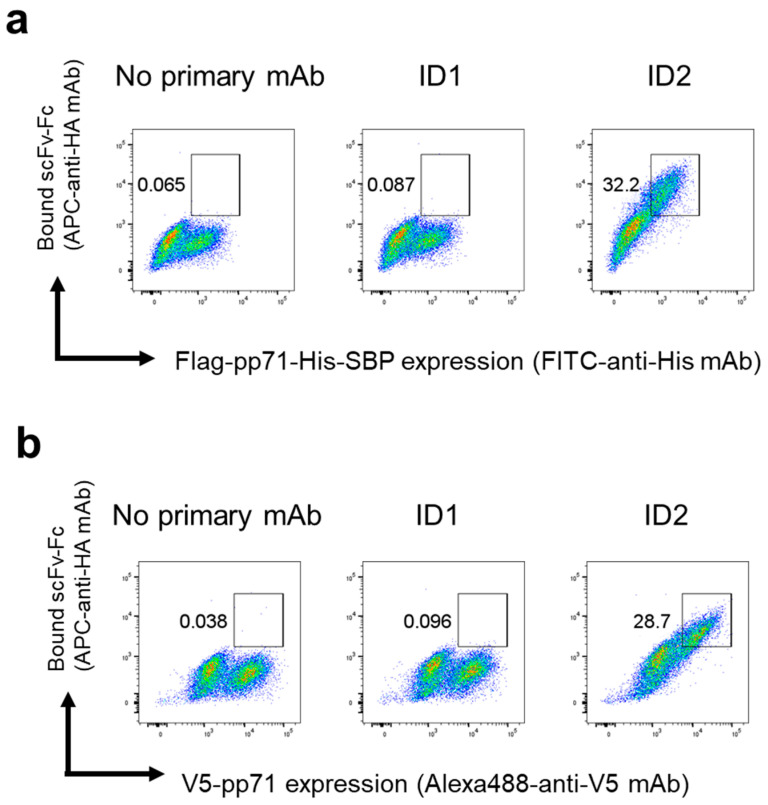
Flow cytometric analysis of anti-pp71 scFv-Fc in HEK293FT cells. (**a**) HEK293FT cells transfected with the Flag-pp71-His-SBP plasmid were stained with FITC-anti-His antibody in the presence of scFv-Fc ID1 or ID2 or without scFv-Fc. The scFv-Fcs were detected using an APC-anti-HA antibody. (**b**) HEK293FT cells transfected with the V5-pp71 plasmid were stained with Alexa488-anti-V5 antibody in the presence of scFv-Fc ID1 or ID2 or without scFv-Fc. The scFv-Fcs were detected using an APC-anti-HA antibody.

We first investigated whether clone ID2 could be used in flow cytometry. An expression plasmid encoding Flag-pp71-His-SBP was transfected into HEK293FT cells. Two days after transfection, the cells were fixed, permeabilized, and then stained with FITC-anti-His antibody, along with the scFv-Fc clone ID1, ID2, or no antibody. As shown in Figure 2a, although ID2 scFv-Fc stained the Flag-pp71-His-SBP-expressing cells, neither ID1 scFv-Fc nor the no-antibody control did. To exclude the possibility that ID2 scFv-Fc might only recognize epitope tags, we transfected HEK293FT cells with a plasmid encoding V5-pp71 and repeated the experiment. As illustrated in Figure 2b, ID2 scFv-Fc also stained V5-pp71-expressing cells, whereas ID1 scFv-Fc and the no-antibody control did not. These results show that ID2 scFv-Fc specifically recognizes pp71 rather than the epitope tags.

**Figure 3:**
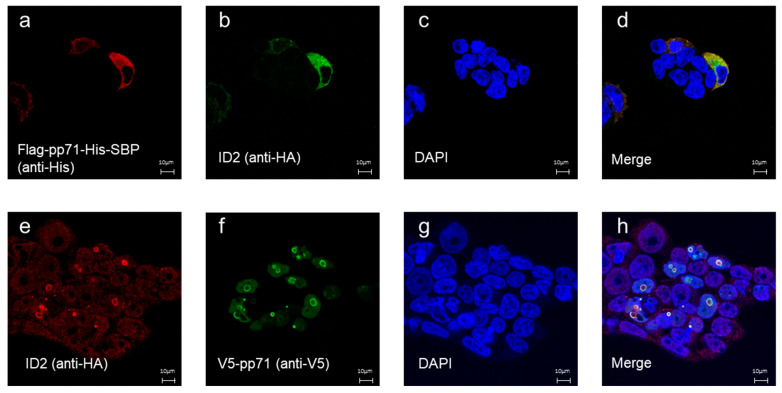
Immunofluorescence analysis using ID2 scFv-Fc in HEK293FT cells. HEK293FT cells transfected with either the Flag-pp71-His-SBP plasmid (**a**–**d**) or V5-pp71 plasmid (**e**–**h**) were incubated with ID2 scFv-Fc and stained with (**a**) Alexa Fluor 594-anti-His tag antibody, (**b**) Alexa Fluor 488-anti-HA tag antibody to detect ID2, (**e**) Alexa Fluor 594-anti-HA tag antibody to detect ID2, (**f**) Alexa Fluor 488-anti-V5 tag antibody, and (**c**,**g**) DAPI. (**d**,**h**) Merged images.

As ID2 scFv-Fc was suitable for flow cytometry, we next examined whether it could be utilized for immunofluorescence staining. It has been reported that pp71 localizes to both the nucleus and cytoplasm, and its overexpression can lead to the formation of nuclear aggregates [8,19,20,21,22]. First, we transfected the Flag-pp71-His-SBP plasmid into HEK293FT cells. After fixation with paraformaldehyde and permeabilization, the cells were stained with ID2 scFv-Fc, anti-His-tag antibody, and DAPI. As shown in Figure 3a-d, the signals of ID2 scFv-Fc and anti-His-tag overlapped, indicating that ID2 scFv-Fc can be used for immunofluorescence.

Interestingly, although unrelated to the inherent function of ID2 scFv-Fc, while Flag-pp71-His-SBP localized in the cytosol, when the V5-pp71 plasmid was transfected and stained with ID2 scFv-Fc and anti-V5 tag antibodies, V5-pp71 mainly localized in nuclear aggregates, indicating that the epitope tags affected pp71 localization (Figure 3e–h).

**Figure 4:**
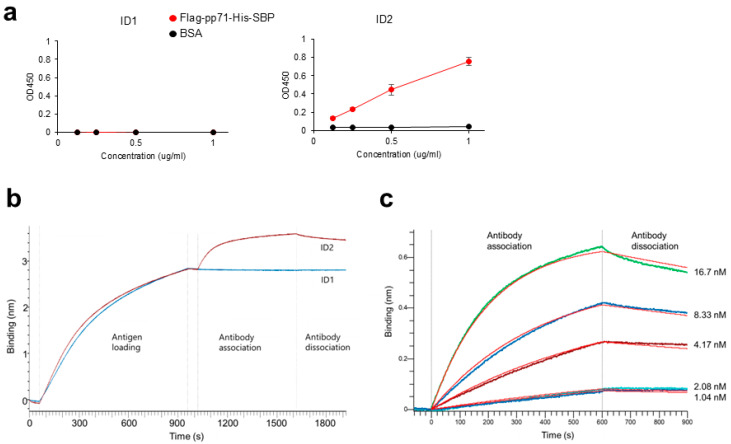
Sandwich ELISA and BLI analyses of anti-pp71 scFv-Fc antibodies. (**a**) Sandwich ELISA. Flag-pp71-SBP-His (red) or BSA (black) on anti-Flag antibody-coated plates were incubated with ID1 or ID2 scFv-Fc, followed by anti-mouse IgG-HRP and TMB substrate. Absorbance was measured using a microplate reader. Data are representative of three independent experiments. Mean  ±  SD of triplicate wells. (**b**) BLI sensorgrams. Real-time binding of ID1 (blue) and ID2 (red) scFv-Fcs to immobilized Flag-pp71-His-SBP is shown. (**c**) Representative graph of BLI sensorgrams of ID2 scFv-Fc binding to immobilized Flag-pp71-His-SBP. Sensorgrams of bold lines indicate different concentrations of ID2 scFv-Fc, and the fitting curves are shown as thin red lines.

Next, we tested whether ID2 scFv-Fc could be used for ELISA. We compared the binding of ID2 scFv-Fc and ID1 scFv-Fc to Flag-pp71-His-SBP or BSA. As shown in Figure 4a, ID2 scFv-Fc bound to Flag-pp71-His-SBP but not to BSA, whereas ID1 scFv-Fc failed to bind to either target. These data demonstrate that ID2 scFv-Fc can also be applied to ELISA.

Finally, we measured the dissociation constant (K_D_) of ID2 scFv-Fc using a biolayer interferometry Octet R4 instrument. Flag-pp71-His-SBP was immobilized on anti-penta-His biosensors, which were then dipped into solutions of ID1 or ID2 scFv-Fc to record association, followed by immersion in buffer alone to record dissociation. As expected, ID1 scFv-Fc did not bind to the antigen, whereas ID2 scFv-Fc showed clear association and dissociation kinetics (Figure 4b). By measuring these kinetics across multiple concentrations of ID2 scFv-Fc (Figure 4c), we determined a K_D_ of 1.316 ± 0.008 nM.

## 4. Discussion

This study reaffirms that a combination of MACS and yeast display technology provides a rapid, low-cost route to obtain high-affinity antibodies [17]. From a human scFv-expressing yeast library, we isolated ID2, which recognizes cytomegalovirus pp71 with sub-nanomolar affinity and performs reliably in immunofluorescence and ELISA assays. Because the relocalization of pp71 is a pivotal trigger of CMV replication [6], ID2 offers a practical tool for dissecting the spatial regulation of the viral life-cycle.

Clinically, pp71 is readily detected by immunohistochemistry in normal prostate epithelium, prostate tumors, and glioblastoma specimen [23,24], highlighting the potential of ID2 for immunohistological analysis of biopsies.

In this study, only twelve individual scFv clones were tested in detail; deep sequencing of the post-selection yeast population would allow for a quantitative view of clonal convergence and might reveal additional anti-pp71 clones that were not recovered in single-colony screening.

Although ID2 itself is intended purely as a research reagent, its discovery highlights the broader translational capability of yeast display. A fully human anti-PD-1 antibody discovered using yeast display technology—sintilimab—received regulatory approval in China in 2018, demonstrating that binders isolated in yeast can meet clinical developability standards without the humanization step demanded by mouse-derived leads [25].

Yeast-derived human scFvs are also advancing into cell therapy: the fully human BCMA-specific CAR CT103A, built with a yeast-selected scFv, achieved a 100% overall response rate and 72% complete/strict complete responses in a phase 1 trial of relapsed/refractory multiple-myeloma patients, with toxicity confined mainly to grade 1–2 cytokine-release syndrome [26].

Together, these examples emphasize that the MACS-plus-yeast display workflow can deliver fully human, high-affinity antibodies that are readily adapted to modern therapeutic formats yet remain accessible to routine academic laboratories. At the same time, its performance is shaped by characteristic strengths and caveats.

Each *S. cerevisiae* cell presents about 3 × 10^4^ scFv molecules on its surface; during magnetic enrichment, this multivalency can capture clones whose intrinsic affinity is weak. A FACS step that simultaneously measures antigen binding and display level removes such avidity-driven binders [13,17,27].

Another limitation of yeast display is library size: yeast display libraries are constrained by transformation efficiencies, commonly achieving practical library diversities of 10^8^ to 10^9^ transformants, considerably smaller than the theoretical diversities attainable with other display technologies such as phage display (10^10^ to 10^12^) and ribosome display (10^12^ to 10^15^) [13,28]

These limitations are balanced by notable advantages. As a eukaryotic host, *S. cerevisiae* supports correct disulfide formation and secretion-quality control, allowing complex antibody fragments to fold properly on the cell surface—an edge over bacterial display systems [14,15]. The platform also enables quantitative, flow-cytometric screening: co-labelling antigen binding and antibody expression permits selection of high-affinity variants normalized for display level [29]. When still greater diversity is required, the gap can be bridged by a hybrid workflow in which a very large phage library is first panned and the enriched pool is subsequently transferred into yeast for FACS-based enrichment [30].

Recognizing both the constraints and unique benefits of the platform will guide its future application to increasingly demanding antiviral and anticancer targets.

## Data Availability

The original contributions presented in this study are included in the article. Further inquiries can be directed to the corresponding author.

## Supplementary Materials

The following supporting information can be downloaded at: https://www.mdpi.com/article/10.3390/antib14030057/s1, Figure S1: SDS-PAGE and CBB staining of purified Flag-pp71-His-SBP; Figure S2: Flow-cytometry analysis of 12 round-5 yeast clones; Table S1: Number of B cells used to construct the human scFv library.

## References

[1] Zuhair M., Smit G.S.A., Wallis G., Jabbar F., Smith C., Devleesschauwer B., Griffiths P. (2019). Estimation of the worldwide seroprevalence of cytomegalovirus: A systematic review and meta-analysis. Rev. Med. Virol..

[2] Fowler K., Mucha J., Neumann M., Lewandowski W., Kaczanowska M., Grys M., Schmidt E., Natenshon A., Talarico C., Buck P.O. (2022). A systematic literature review of the global seroprevalence of cytomegalovirus: Possible implications for treatment, screening, and vaccine development. BMC Public Health.

[3] Ramanan P., Razonable R.R. (2013). Cytomegalovirus infections in solid organ transplantation: A review. Infect. Chemother..

[4] Schleiss M.R. (2013). Cytomegalovirus in the neonate: Immune correlates of infection and protection. Clin. Dev. Immunol..

[5] Griffiths P., Reeves M. (2021). Pathogenesis of human cytomegalovirus in the immunocompromised host. Nat. Rev. Microbiol..

[6] Kalejta R.F. (2008). Tegument proteins of human cytomegalovirus. Microbiol. Mol. Biol. Rev..

[7] Saffert R.T., Kalejta R.F. (2006). Inactivating a cellular intrinsic immune defense mediated by Daxx is the mechanism through which the human cytomegalovirus pp71 protein stimulates viral immediate-early gene expression. J. Virol..

[8] Trgovcich J., Cebulla C., Zimmerman P., Sedmak D.D. (2006). Human cytomegalovirus protein pp71 disrupts major histocompatibility complex class I cell surface expression. J. Virol..

[9] Kalejta R.F., Bechtel J.T., Shenk T. (2003). Human cytomegalovirus pp71 stimulates cell cycle progression by inducing the proteasome-dependent degradation of the retinoblastoma family of tumor suppressors. Mol. Cell Biol..

[10] Cimato G., Zhou X., Brune W., Frascaroli G. (2024). Human cytomegalovirus glycoprotein variants governing viral tropism and syncytium formation in epithelial cells and macrophages. J. Virol..

[11] Nowak B., Sullivan C., Sarnow P., Thomas R., Bricout F., Nicolas J.C., Fleckenstein B., Levine A.J. (1984). Characterization of monoclonal antibodies and polyclonal immune sera directed against human cytomegalovirus virion proteins. Virology.

[12] Köhler G., Milstein C. (1975). Continuous cultures of fused cells secreting antibody of predefined specificity. Nature.

[13] Hoogenboom H.R. (2005). Selecting and screening recombinant antibody libraries. Nat. Biotechnol..

[14] Boder E.T., Wittrup K.D. (1997). Yeast surface display for screening combinatorial polypeptide libraries. Nat. Biotechnol..

[15] Angelini A., Chen T.F., de Picciotto S., Yang N.J., Tzeng A., Santos M.S., Van Deventer J.A., Traxlmayr M.W., Wittrup K.D. (2015). Protein Engineering and Selection Using Yeast Surface Display. Methods Mol. Biol..

[16] Benatuil L., Perez J.M., Belk J., Hsieh C.M. (2010). An improved yeast transformation method for the generation of very large human antibody libraries. Protein Eng. Des. Sel..

[17] Chao G., Lau W.L., Hackel B.J., Sazinsky S.L., Lippow S.M., Wittrup K.D. (2006). Isolating and engineering human antibodies using yeast surface display. Nat. Protoc..

[18] Wentz A.E., Shusta E.V. (2007). A novel high-throughput screen reveals yeast genes that increase secretion of heterologous proteins. Appl. Environ. Microbiol..

[19] Marshall K.R., Rowley K.V., Rinaldi A., Nicholson I.P., Ishov A.M., Maul G.G., Preston C.M. (2002). Activity and intracellular localization of the human cytomegalovirus protein pp71. J. Gen. Virol..

[20] Prichard M.N., Sztul E., Daily S.L., Perry A.L., Frederick S.L., Gill R.B., Hartline C.B., Streblow D.N., Varnum S.M., Smith R.D. (2008). Human cytomegalovirus UL97 kinase activity is required for the hyperphosphorylation of retinoblastoma protein and inhibits the formation of nuclear aggresomes. J. Virol..

[21] Hensel G.M., Meyer H.H., Buchmann I., Pommerehne D., Schmolke S., Plachter B., Radsak K., Kern H.F. (1996). Intracellular localization and expression of the human cytomegalovirus matrix phosphoprotein pp71 (ppUL82): Evidence for its translocation into the nucleus. J. Gen. Virol..

[22] Shen W., Westgard E., Huang L., Ward M.D., Osborn J.L., Chau N.H., Collins L., Marcum B., Koach M.A., Bibbs J. (2008). Nuclear trafficking of the human cytomegalovirus pp71 (ppUL82) tegument protein. Virology.

[23] Classon J., Stenudd M., Zamboni M., Alkass K., Eriksson C.J., Pedersen L., Schorling A., Thoss A., Bergh A., Wikstrom P. (2025). Cytomegalovirus infection is common in prostate cancer and antiviral therapies inhibit progression in disease models. Mol. Oncol..

[24] Matlaf L.A., Harkins L.E., Bezrookove V., Cobbs C.S., Soroceanu L. (2013). Cytomegalovirus pp71 protein is expressed in human glioblastoma and promotes pro-angiogenic signaling by activation of stem cell factor. PLoS ONE.

[25] Deng M. (2019). The approval of sintilimab for classical Hodgkin’s lymphoma: Views and perspectives of anti-PD-1/PD-L1 antibodies in China. Antib. Ther..

[26] Wang D., Wang J., Hu G., Wang W., Xiao Y., Cai H., Jiang L., Meng L., Yang Y., Zhou X. (2021). A phase 1 study of a novel fully human BCMA-targeting CAR (CT103A) in patients with relapsed/refractory multiple myeloma. Blood.

[27] Lopez-Morales J., Vanella R., Kovacevic G., Santos M.S., Nash M.A. (2023). Titrating Avidity of Yeast-Displayed Proteins Using a Transcriptional Regulator. ACS Synth. Biol..

[28] Slavny P., Hegde M., Doerner A., Parthiban K., McCafferty J., Zielonka S., Hoet R. (2024). Advancements in mammalian display technology for therapeutic antibody development and beyond: Current landscape, challenges, and future prospects. Front. Immunol..

[29] Wang B., DeKosky B.J., Timm M.R., Lee J., Normandin E., Misasi J., Kong R., McDaniel J.R., Delidakis G., Leigh K.E. (2018). Functional interrogation and mining of natively paired human VH:VL antibody repertoires. Nat. Biotechnol..

[30] Bidlingmaier S., Su Y., Liu B. (2015). Combining Phage and Yeast Cell Surface Antibody Display to Identify Novel Cell Type-Selective Internalizing Human Monoclonal Antibodies. Methods Mol. Biol..

