# Supplementary material for: Isolation of a Monoclonal Human scFv Against Cytomegalovirus pp71 Antigen Using Yeast Display
Source: Antibodies (Basel). 2025 Jul 10;14(3):57. doi: 10.3390/antib14030057 (PMC12286267; doi:10.3390/antib14030057)
Supplement: Supplementary file 1 [file antibodies-14-00057-s001.zip › Supplementary Figure Legend.pdf]

Supplementary Figure Legend

Supplementary Figure S1. CBB staining of purified Flag-pp71-His-SBP. Indicated amount of Flag-pp71-His-SBP was subjected to SDS-PAGE, followed by CBB staining. Estimated Flag-pp71-His-SBP molecular weight was 68.3 kDa.

Supplementary Figure S2. Flow cytometry plots showing Flag-pp71-His-SBP binding to yeast cells derived from single colonies in round 5. Sanger sequencing revealed that clones A, E, H, and K shared an identical scFv sequence (ID2), whereas clone C possessed a distinct sequence (ID1).
