# Supplementary figures and images for: Isolation of a Monoclonal Human scFv Against Cytomegalovirus pp71 Antigen Using Yeast Display
Source: Antibodies (Basel). 2025 Jul 10;14(3):57. doi: 10.3390/antib14030057 (PMC12286267; doi:10.3390/antib14030057)

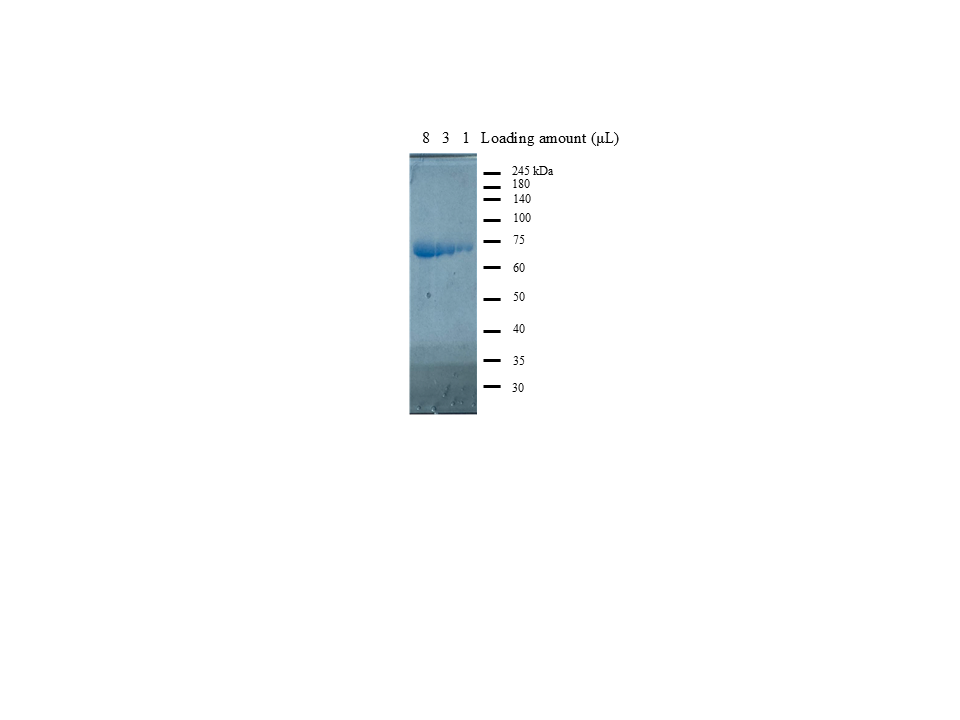

Supplement: Supplementary file 1 [file antibodies-14-00057-s001.zip › Supplementary Figure S1.tif]

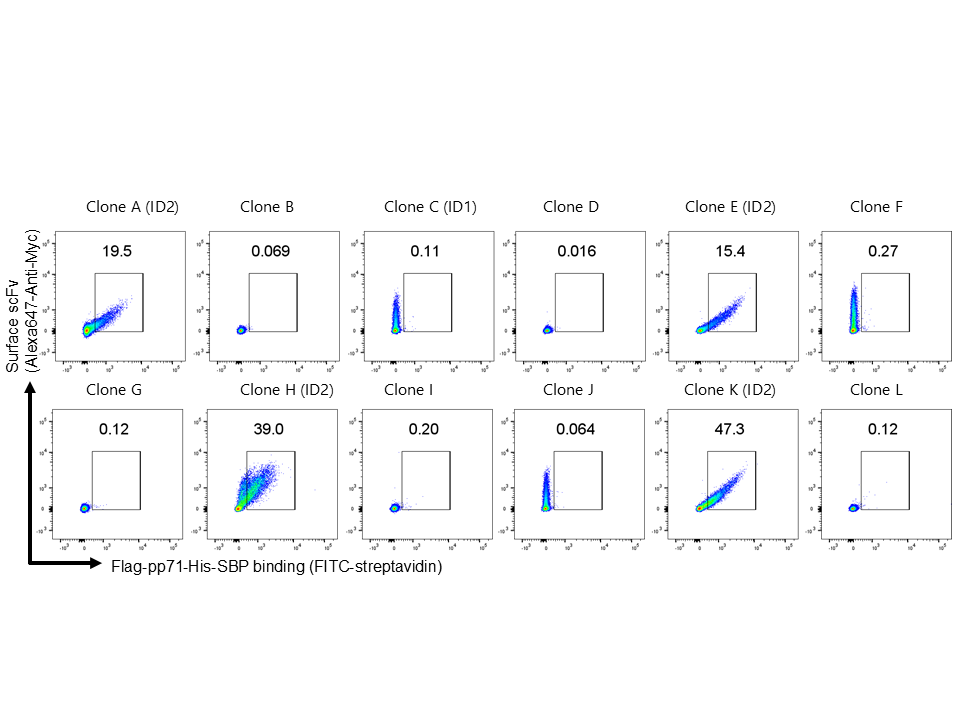

Supplement: Supplementary file 1 [file antibodies-14-00057-s001.zip › Supplementary Figure S2.tif]
